# Cost-Effectiveness of Pembrolizumab for the treatment of Non–Small-Cell lung cancer: A systematic review

**DOI:** 10.3389/fonc.2022.815587

**Published:** 2022-08-26

**Authors:** Chuan Zhang, Jiaxu Zhang, Jing Tan, Panwen Tian, Weimin Li

**Affiliations:** ^1^ Department of Pharmacy, West China Second University Hospital, Sichuan University, Chengdu, China; ^2^ Evidence-Based Pharmacy Center, West China Second University Hospital, Sichuan University, Chengdu, China; ^3^ Key Laboratory of Birth Defects and Related Diseases of Women and Children (Sichuan University), Ministry of Education, Chengdu, China; ^4^ West China School of Pharmacy, Sichuan University, Chengdu, China; ^5^ Chinese Evidence-based Medicine Center, West China Hospital, Sichuan University, Chengdu, China; ^6^ Department of Respiratory Medicine, West China Hospital, Sichuan University, Chengdu, China

**Keywords:** immune checkpoint inhibitors, pembrolizumab, cost-effectiveness, systematic review, pharmacoeconomic

## Abstract

**Introduction:**

Pembrolizumab, an immune checkpoint inhibitor for treating non-small cell lung cancer (NSCLC), can impose a high financial burden. Several studies have explored the cost-effectiveness of this expensive agent. We conducted a systematic review and pooled analysis to evaluate the quality of the existing pharmacoeconomic studies on pembrolizumab strategies for NSCLC treatment as well as to conclude the cost-effectiveness of such strategies.

**Methods:**

English and Chinese databases were searched to collect health economic studies on pembrolizumab therapies (monotherapy or a combination with chemotherapy) compared with chemotherapy for the treatment of NSCLC patients. The reporting quality, modeling methods, and results of incremental cost-effectiveness analysis of the included literature were descriptively analyzed.

**Results:**

A total of 24 studies, 3 in Chinese and 21 in English, were selected. All reports satisfy a median of 31 out of 40 reporting quality assessment items based on a quality checklist for pharmacoeconomic evaluations. 12 studies used the Markov model and 11 used the partitioned survival model. A common problem identified in the modeling methods was the insufficient justification of the choices of model structure and data inputs. Pembrolizumab was found to be cost-effective in the United States and Switzerland, but not in China, France, the UK, or Singapore.

**Conclusion:**

The current cost-effectiveness studies on pembrolizumab for the treatment of NSCLC are of moderate quality, and the relevant decision-analytic modeling methods have much scope for improvement. The cost-effectiveness of pembrolizumab strategies for NSCLC varies across countries, warranting the need to pay more attention to the methodologies of pharmacoeconomic research in order to produce correct outcomes in terms of cost-effectiveness for different countries.

**Systematic Review Registration:**

https://www.crd.york.ac.uk/PROSPERO/, identifier CRD42021250480

## Introduction

Lung cancer is a type of malignant tumor with the highest morbidity and mortality among all cancer types. In 2020 alone, it contributed to 1.8 million out of 9.96 million cancer-related deaths globally (1). In histopathological terms, lung cancer can be classified as small cell lung cancer (SCLC) or non-small cell lung cancer (NSCLC), representing approximately 15% and 85% of all lung cancer cases, respectively (2). Owing to the relatively low diagnosis rate of NSCLC at the early stages, approximately 17.6% of NSCLC were diagnosed at stage IIIB and 40% at stage IV (3), with an estimated 5-year survival rate to be 16% (4). In patients receiving the conventional platinum-based doublet chemotherapy for advanced NSCLC without driver gene mutations, the median overall survival was <1 year (5), implicating the urgent need for improving the therapeutic efficacy of current treatment options.

Immune checkpoint inhibitors (ICIs) have made groundbreaking progress in the treatment of cancer. Currently, the US Food and Drug Administration (FDA) has approved 6 PD-1/PD-L1 inhibitors (6). Several clinical trials (7–13) have presented shown evidence that pembrolizumab immunotherapy when compared with chemotherapy, can significantly improve the clinical outcomes for NSCLC patients. In October 2016, based on 2 randomized controlled trials (RCTs), KEYNOTE-010 (7) and KEYNOTE-024 (8), pembrolizumab was approved by the FDA for the treatment of metastatic NSCLC patients identified as PD-L1 positive, that is, with a PD-L1 Tumor Expression Score (TPS) ≥1% as determined by an FDA-approved test. For the first time, an ICI was approved as the first-line treatment of lung cancer, with an extension of indications of pembrolizumab from high PD-L1 expression (TPS ≥50%) to PD-P1 positive (TPS ≥1%).

As a novel treatment option, the high costs of ICIs can place a heavy burden on society, as the cost-effectiveness of such therapies remains debatable. This study aimed to conclude, through a systematic review of health economic studies relevant to pembrolizumab for the treatment of NSCLC, the cost-effectiveness of pembrolizumab as a single agent or in combination with chemotherapy, relative to the standard or conventional chemotherapy, thereby facilitating and providing evidence for healthcare resource allocation and policy-making regarding the treatment of NSCLC with pembrolizumab.

## Methods

### Literature search

Past studies were searched and screened in adherence to the PRISMA guidelines (14). We searched 7 databases, including the English databases Cochrane Library, Embase, PubMed, and EBSCO EconLit, and Chinese databases VIP, Wanfang Database, and CNKI to collect studies published from database inception until March 26, 2021. The details of searching strategies are presented in **Appendix A.1**. After the removal of duplicates, the studies were preliminarily screened by examining the titles and abstracts based on pre-specified inclusion and exclusion criteria. The studies included after the preliminary screening were secondarily screened by reading the full text to identify studies that could be included in the final analysis.

### Inclusion and exclusion

The search results were selected in accordance with the following inclusion criteria: (1) patients with NSCLC were the target population; (2) pembrolizumab monotherapy or pembrolizumab combined with chemotherapy was used as the intervention strategy; (3) chemotherapy drugs were used as the comparator; (4) studies that report pharmacoeconomic outcomes, including cost, life-years (LY), quality-adjusted life-years (QALY), and incremental cost-effectiveness ratio (ICER); and (5) the study design was economic evaluations (cost-effectiveness, cost-benefit and cost-utility analyses). Studies were excluded if they were: (1) without any decision-analytic model; (2) without clinical data inputs from any clinical trial; (2) duplicates; (3) conference abstracts, editorials, literature reviews, comments, notes, or letters; (4) not written in Chinese or English; or (5) unavailable for full-text after active measures were taken to acquire access.

### Assessment of reporting quality

The reporting quality of the included health economic studies was assessed with reference to a quality assessment checklist described by *The China Guidelines for Pharmacoeconomic Evaluations 2020 Edition* (15). This checklist is based on the guideline’s recommended standard reporting format and contains areas specific to health economic evaluations, for example, evaluation of cost and utility data sources. We adapted the checklist to 40 yes/no questions (**Appendix A.2**) suitable for the assessment of reporting quality. A study was assessed based on whether it met the quality requirement proposed by each question unless the question was not applicable. Accordingly, the quality of a study was determined by the number of checklist questions whose requirements were satisfied by the study, with a larger number representing higher quality.

### Assessment of modeling methods

We examined the modeling methods used in the studies with regard to the structures and input parameters of the decision-analytic models. The structural assumptions were examined in terms of the research perspectives, model types, health states, and transitions among them, modeling cycles, and time horizons. The sources of cost, utility, and clinical efficacy inputs, as well as the means of their estimation and analysis, were also evaluated. In additional, results from the uncertainty analyses in each study are also reported to evaluate whether parameter uncertainties, structural uncertainties, and methodological uncertainties of the models were appropriately addressed.

### Evaluation of cost-effectiveness results

We compared the results and conclusions from the included cost-effectiveness analysis (CEA) on pembrolizumab monotherapy or pembrolizumab combined with chemotherapy, relative to chemotherapy, to ascertain the cost-effectiveness of pembrolizumab regimens in different countries. Furthermore, we compared the ICER results from different studies that shared similar country perspectives and clinical data sources to explore the potential factors, e.g. model structures and parameter inputs, that might contribute to the different outcomes.

## Results

### Results of literature selection

**Figure 1:**
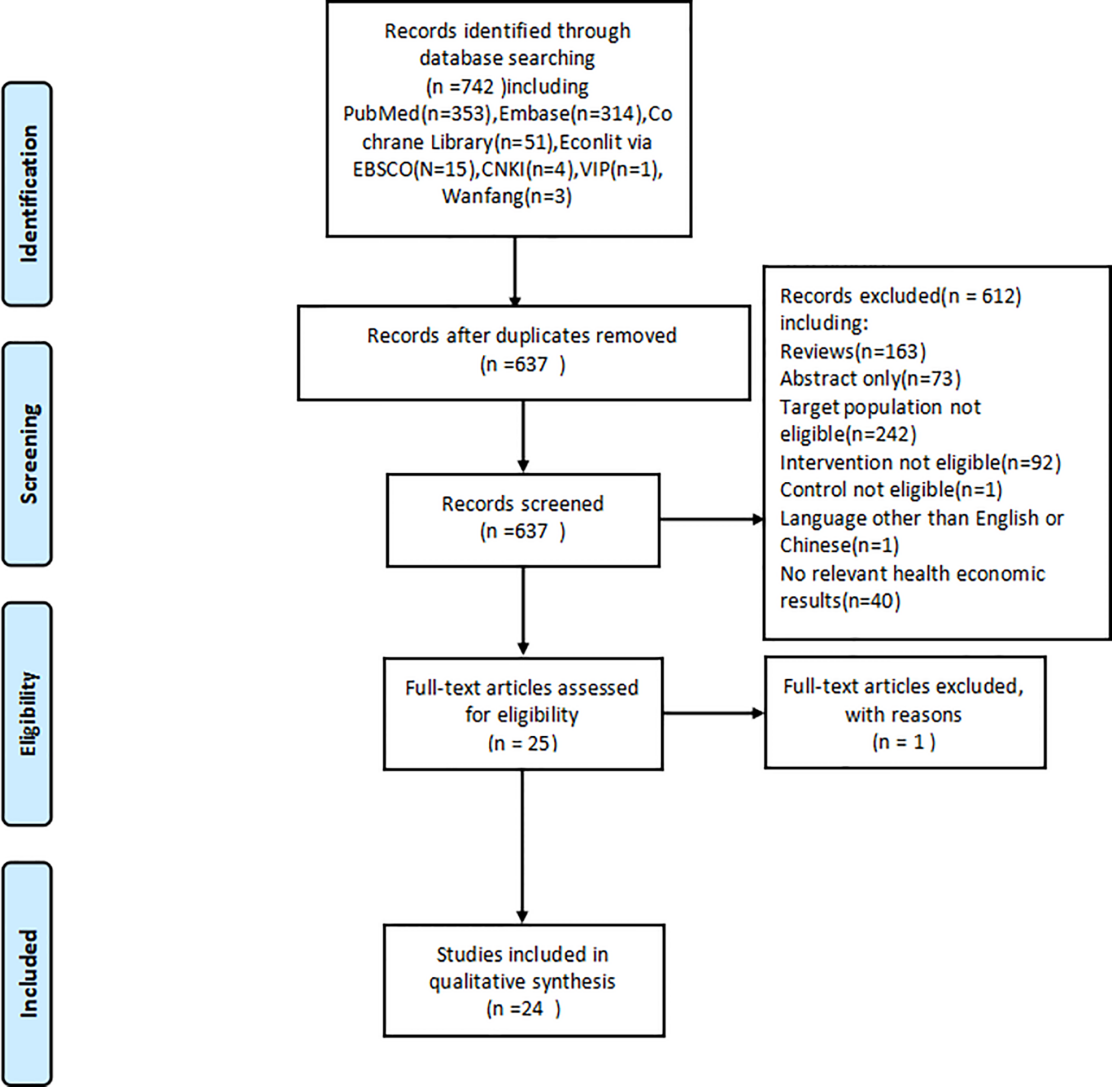
Search flow chart.

A total of 742 studies were collected after the database search. After the removal of duplicates, 637 studies entered the preliminary screening, wherein each study was assessed with reference to the inclusion and exclusion criteria through an examination of their titles and abstracts. 24 eligible studies (16–39) were identified in the primary screening and further examined *via* secondary screening by reading the full text, whereby all 24 studies were included in the final analysis. The process of searching and screening is illustrated in **Figure 1**.

The 24 studies for the final analysis included three in Chinese and 21 in English languages; these studies were published in the recent 5 years, with the earliest record published in 2017 and 20 records during 2019–2021. All included studies were based on model-based CEA of pembrolizumab in the treatment of NSCLC, using clinical data from one or more clinical trials. Five trials related to pembrolizumab, namely, KEYNOTE-010 (7), KEYNOTE-024 (8), KEYNOTE-189 (10), KEYNOTE-407 (11), and KEYNOTE-042 (13), were referred to as clinical data sources by these studies. For the base-case analysis, 7 studies investigated the cost-effectiveness of pembrolizumab combined with chemotherapy as the intervention strategy of interest, whereas 15 studies investigated pembrolizumab monotherapy, with conventional or standard chemotherapy as the comparator. For ICER outcomes, 14 studies reported outcomes as incremental cost per QALY, whereas 10 studies reported both incremental costs per QALY and per LY. 21 studies were from a single country’s perspective, and 3 studies conducted the CEA in two country settings. The United States (US) and China were the two most common settings, with 12 and 9 analyses, respectively. The other country settings were Switzerland, France, the United Kingdom (UK), and Singapore. Most studies in the US setting were from the perspective of third-party payers, whereas the other studies were mainly perspectives of society or healthcare systems. The basic information of the included studies is summarized in **Table 1**.

**Table 1:** Key study information & reporting quality.

Reference	Clinical Data Source	Population	Intervention	Comparator	Outcomes	Country Background	Reporting Quality (Out of 40 Items)
**Lei (** 16)	KN189	Previously untreated advanced non-squamous NSCLC without EGFR or ALK mutations	Pembrolizumab + PT-Chem	PT-Chem	Incremental cost/QALY	China	25
**Jiang (** 17)	KN189	Previously untreated advanced non-squamous NSCLC without EGFR or ALK mutations	Pembrolizumab + PT-Chem	PT-Chem	Incremental cost/QALYIncremental cost/LY	China	34
**Zeng (** 18)	KN189	Previously untreated advanced non-squamous NSCLC without EGFR or ALK mutations	Pembrolizumab + PT-Chem	PT-Chem	Incremental cost/QALY	US	32
**Insinga (** 19)	KN189	Previously untreated advanced non-squamous NSCLC without EGFR or ALK mutations	Pembrolizumab + PT-Chem	PT-Chem	Incremental cost/QALYIncremental cost/LY	US	34
**Wan (** 20)	KN189	Previously untreated advanced non-squamous NSCLC without EGFR or ALK mutations	Pembrolizumab + PT-Chem	PT-Chem	Incremental cost/QALY	US & China	29
**Wu (** 21)	KN407, KN189	Previously untreated metastatic squamous and non-squamous NSCLC without EGFR or ALK mutations	Pembrolizumab + PT-Chem	PT-Chem	Incremental cost/QALY	US & China	32
**Insinga (** 22)	KN407	Previously untreated metastatic squamous NSCLC without EGFR or ALK mutations	Pembrolizumab + PT-Chem	PT-Chem	Incremental cost/QALYIncremental cost/LY	US	34
**Barbier (** 23)	KN024	Previously untreated stage IV (mostly non-squamous) NSCLC with PD-L1 expression ≥ 50% and without EGFR or ALK mutations	Pembrolizumab monotherapy	PT-Chem	Incremental cost/QALYIncremental cost/LY	Switzerland	29
**Bhadhur (** 24)	KN024	Previously untreated stage IV NSCLC with ≥ 50% PD-L1 expression, without EGFR or ALK mutations	Pembrolizumab monotherapy	PT-Chem	Incremental cost/QALYIncremental cost/LY	Switzerland	32
**Hu (** 25)	KN024	Previously untreated stage IV NSCLC with ≥ 50% PD-L1 expression and without EGFR or ALK mutations	Pembrolizumab monotherapy	PT-Chem	Incremental cost/QALY	UK	30
**Georgieva (** 26)	KN024	Previously untreated stage IV NSCLC with ≥ 50% PD-L1 expression and without EGFR or ALK mutations	Pembrolizumab monotherapy	PT-Chem	Incremental cost/QALY	US & UK	29
**Huang (** 27)	KN024	Previously untreated stage IV NSCLC with ≥ 50% PD-L1 expression, without EGFR or ALK mutations	Pembrolizumab monotherapy	PT-Chem	Incremental cost/QALYIncremental cost/LY	US	32
**Liao (** 28)	KN024	Previously untreated stage IV NSCLC with ≥ 50% PD-L1 expression and without EGFR or ALK mutations	Pembrolizumab monotherapy	PT-Chem	Incremental cost/QALY	China	30
**Loong (** 29)	KN024	Previously untreated stage IV NSCLC with ≥ 50% PD-L1 expression, without EGFR or ALK mutations	Pembrolizumab monotherapy	PT-Chem	Incremental cost/QALYIncremental cost/LY	China (Hong Kong)	29
**Chouaid (** 30)	KN024	Previously untreated stage IV NSCLC with ≥ 50% PD-L1 expression, without EGFR or ALK mutations	Pembrolizumab monotherapy	PT-Chem	Incremental cost/QALYIncremental cost/LY	France	33
**Aziz (** 31)	KN024	Previously untreated stage IV NSCLC with ≥ 50% PD-L1 expression, without EGFR or ALK mutations	Pembrolizumab monotherapy	PT-Chem	Incremental cost/QALY	Singapore	35
**She (** 32)	KN042	Previously untreated locally advanced NSCLC with ≥ 1% PD-L1 expression, without EGFR or ALK mutations	Pembrolizumab monotherapy	PT-Chem	Incremental cost/QALY	US	31
**Weng (** 33)	KN042	Previously untreated locally advanced NSCLC with ≥ 1% PD-L1 expression, without EGFR or ALK mutations	Pembrolizumab monotherapy	PT-Chem	Incremental cost/QALY	US	31
**Huang (** 34)	KN042	Previously untreated locally advanced NSCLC with ≥ 1% PD-L1 expression, without EGFR or ALK mutations	Pembrolizumab monotherapy	PT-Chem	Incremental cost/QALYIncremental cost/LY	US	35
**Zhou (** 35)	KN042	Previously untreated locally advanced NSCLC with ≥ 1% PD-L1 expression, without EGFR or ALK mutations	Pembrolizumab monotherapy	PT-Chem	Incremental cost/QALY	China	27
**Xu (** 36)	KN042	Previously untreated locally advanced NSCLC with ≥ 1% PD-L1 expression, without EGFR or ALK mutations	Pembrolizumab monotherapy	PT-Chem	Incremental cost/QALY	China	30
**Xu (** 37)	KN042	Previously untreated locally advanced NSCLC with ≥ 1% PD-L1 expression, without EGFR or ALK mutations	Pembrolizumab monotherapy	PT-Chem	Incremental cost/QALY	China	29
Huang (38)	KN010	Second-line NSCLC with PD-L1 expression ≥ 1%	Pembrolizumab monotherapy	Docetaxel	Incremental cost/QALYIncremental cost/LY	US	32
**Aguiar (** 39)	KN010	Second-line NSCLC with PD-L1 expression ≥ 1%	Pembrolizumab monotherapy	Docetaxel	Incremental cost/QALY	US	24

ALK, Anaplastic lymphoma kinase; EGFR, epidermal growth factor receptor; KN, KEYNOTE; LY, life-year; NSCLC, non-small cell lung cancer; PT-C, platinum-based chemotherapy; QALY, quality-adjusted life-year; US, the United States; UK, the United Kingdom.

### Results of literature quality assessment

By the reporting quality assessment checklist described in **Appendix A.2**; the quality of the studies varied as the number of items met by each study ranged from 24 to 35 (median 31). Specifically, 2 studies satisfied ≤25 items in the checklist, 9 studies satisfied 26-30 items, and 14 studies between 31-35 items. The three reports written in Chinese satisfied an average of 28 items, and the English reports satisfied an average of 31.4 items. None of the studies were able to fully meet the reporting quality assessment criteria. Three studies that satisfied the requirements of 35 checklist questions, which were the highest among the included studies, did not meet the requirements in certain aspects. These included a lack of a systematic literature review, ambiguous description of the research question, or insufficient justification for the choice of the comparators. The checklist questions represented by these aspects were also among the least satisfied items by all the included studies. The results of the literature reporting quality assessment are presented in **Appendix A.3** and summarized in the last column of **Table 1**.

### Results of modeling method analysis

#### Model structure assumptions

The Markov model was used in 12 of the 24 studies, some with a combined decision tree, and the partitioned survival (PS) model was utilized in 11 studies. In one of the studies, the model type was not specified. In most model structures, three health states representing progression-free survival (PFS), disease progression (PD), and death were adopted. Georgieva et al. (26) presented a total of five health states by adding two absorption states (treatment termination due to related AEs and treatment termination due to PD) in addition to death. The extracted information on modeling methods is presented in **Table 2**.

**Table 2:** Key information of model structures and parameter inputs.

Reference	Model Type	Time Horizon	Modeling cycle	Parametric distributions for extrapolation of survival in the base-case	Type of costs	Inclusion of adverse events	Approach to Utilities	Annual discount rate for cost and outcome
**Lei (** 16)	Markov	10 years	21 days	Unspecified	Direct medical costs	AEs of grade 3-4	By health states	5%, unspecified whether it is applied to cost or outcome, or both
**Jiang (** 17)	PS	20 years	7 days	PFS experimental arm: Weibull and log-normal; PFS control arm: Weibull; OS in both arms: exponential distribution	Direct medical costs	AEs of grade ≥ 3	By time-to-death	3%
**Zeng (** 18)	Markov	20 years	21 days	Exponential distribution for OS and Weibull for PFS in both groups	Direct medical costs	AEs of grade ≥ 3 with incidence ≥ 5%	By health states; disutility of AEs considered	3%
**Insinga (** 19)	PS	20 years	7 days	ToT: exponential distribution for experimental arm, Gompertz for control arm; PFS experimental arm: K-M data till Week 39, Weibull thereafter; PFS control arm: K-M data till Week 21, Weibull thereafter; OS data in both arms, K-M data till Week 31, exponential distribution thereafter	Direct medical costs	AEs of grade ≥ 3 with incidence ≥ 5%	By time-to-death	3%
**Wan (** 20)	Markov	Lifetime	21 days	Weibull and log-logistic	Direct medical costs	AEs of grade ≥ 3	By health states	3%
**Wu (** 21)	Decision Tree + Markov	20 years	21 days	Royston/Parmar model for pooled OS of two trials; log-normal model for PFS of KN189; log-logistic model for PFS of KN407	Direct medical costs	AEs of grade ≥ 3	By time-to-death	3% (US) 5% (CN)
**Insinga (** 22)	PS	20 years	7 days	ToT: gen-gamma for experimental arm, original K-M for control arm; PFS in both arms: K-M data till Week 26, log-normal thereafter; OS in both arms: K-M data till Week 19, exponential distribution thereafter	Direct medical costs	AEs of grade ≥ 3 with incidence ≥ 5%	By time-to-death	3%
**Barbier (** 23)	Markov	10 years	30 days	Exponential distribution for OS and lognormal distribution for PFS in both groups	Direct medical costs	AEs of grade 3-4	By health states and treatment arms	3%
**Bhadhur (** 24)	PS	20 year	7 days	ToT: Weibull for experimental arm, gen-gamma for control arm. PFS experimental arm: K-M data till Week 9, Weibull thereafter. PFS control arm: K-M data till Week 9, exponential distribution thereafter. OS: K-M till Week 32 for experimental arm and Week 38 for control arm, exponential distribution for both arms from then to Year 5, a constant mortality rate from Year 5 to 20	Direct medical costs	AEs of grade ≥ 3 with incidence ≥ 5% + pneumonitis	By time-to-death	3%
**Hu (** 25)	Markov	Lifetime (until 99% patients have died)	21 days	Unspecified	Direct medical costs	AEs of grade ≥ 3 with incidence ≥ 5%	By health states and treatment arms; disutility of AEs considered	3.5%
**Georgieva (** 26)	Bayesian Markov	Lifetime	30 days	Weibull for OS and PFS in both groups	Direct medical costs	Included AEs are listed, without inclusion criteria specified	By health states	No discounting in the base-case; 3% in scenario analysis
**Huang (** 27)	PS	20 years	7 days	ToT: Weibull for experimental arm, gen-gamma for control arm; PFS experimental arm: K-M data till Week 9, Weibull thereafter. PFS control arm: K-M data till Week 9, exponential distribution thereafter. OS: K-M till Week 32 for experimental arm and Week 38 for control arm, exponential distribution for both arms from then to Year 5, a constant mortality rate from Year 5 to 20	Direct medical costs	AEs of grade ≥ 3 with incidence ≥ 5% + pneumonitis	By time-to-death	3%
**Liao (** 28)	Markov	10 years	30 days	Unspecified	Direct medical costs	Unspecified	By health states	3%
**Loong (** 29)	PS	10 years	7 days	PFS: K-M data for both arms till Week 9, Weibull for experimental arm and exponential distribution for control arm thereafter. OS experimental arm: K-M data till Week 32, exponential distribution from Week 32 to Year 5; OS control arm: K-M data till Week 38, exponential distribution from Week 38 to Year 5, a constant mortality rate from Year 5 to 20 for both arms	Direct medical costs	AEs of grade ≥ 3 with incidence ≥ 5% + pneumonitis	By time-to-death and health states	3%
**Chouaid (** 30)	PS	10 years	7 days	PFS in both arms: K-M data till Week 9, Weibull and exponential distribution for squamous NSCLC, gen-gamma and exponential distribution for non-squamous NSCLC thereafter; OS experimental arm, K-M data till Week 22, exponential distribution thereafter; OS control arm: K-M data till Week 15, exponential distribution thereafter	Direct medical costs	AEs of grade ≥ 3 with incidence ≥ 1%, excluding diabetes mellitus	By health states; disutility of AEs considered	4%
**Aziz (** 31)	PS	10 years	7 days	OS in both arms: K-M data till Week 33, exponential distribution thereafter; PFS in both arms: K-M data till Week 9, Weibull thereafter	Direct medical costs	AEs of grade ≥ 3	By health states and lines of therapy; disutility of AEs considered	3%
**She (** 32)	Decision tree + Markov	20 years	42 days	Weibull for OS and PFS in both groups	Direct medical costs	AEs of grade ≥ 3 with incidence ≥ 5%	By health states and treatment arms	3%
**Weng (** 33)	Markov	Lifetime (until 99% patients have died)	21 days	Weibull for OS and PFS in both groups	Direct medical costs	Four kinds of SAEs with incidence ≥ 5%	By health states and treatment arms	3% discount rate for costs, unspecified for outcomes
**Huang (** 34)	PS	20 years	Unspecified	ToT: K-M data; PFS in both arms: K-M data till Week 9, Weibull for experimental arm and exponential distribution for control arm thereafter; OS in both arms: K-M data till Week 33, exponential distribution from then to Year 4, a constant mortality rate from Year 4 to 20.	Direct medical costs	AEs of grade 3-5	By time-to-death	3%
**Zhou (** 35)	Markov	10 years	21 days	Unspecified	Direct medical costs	AEs of grade ≥ 3	By health states	3%
**Xu (** 36)	Markov	Lifetime	21 days	OS regardless of TPS: Gen-gamma for experimental arm, log-logistic for control arm; PFS TPS ≥50%: Log-normal for experimental arm, gen-gamma for control arm; PFS TPS ≥20%: gen-gamma for experimental arm, log-logistic for control arm; PFS TPS ≥1%: log-logistic for experimental arm, gen-gamma for control arm	Direct medical costs	AEs of grade ≥ 3 with incidence ≥ 3%	By health states	5%
**Xu (** 37)	PS	20 years	21 days	OS regardless of TPS: Gen-gamma for experimental arm, log-logistic for control arm; PFS experimental arm: log-normal (TPS ≥ 50%), gen-gamma (TPS ≥ 20%), log-logistic (TPS ≥ 1%); PFS control arm: gen-gamma (TPS ≥ 50% and TPS ≥ 1%), log-logistic (TPS ≥ 20%)	Direct medical costs	AEs of grade ≥ 3 with incidence ≥ 3%	By health states	5%
Huang (38)	PS	20 years	7 days	ToT: Gompertz for experimental arm, K-M data for control arm; PFS in both arms: K-M data till Week 9, Weibull for experimental arm and exponential distribution for control arm thereafter; OS in both arms: K-M data till Week 52, exponential distribution from then to Year 5, a constant mortality rate from Year 5 to 20.	Direct medical costs	AEs of grade ≥ 3 with incidence ≥ 5%	By time-to-death; disutility of AEs considered	3%
**Aguiar (** 39)	Unspecified model type	5 years	Unspecified	Unspecified	Direct medical costs	Unspecified	By health states	No discount in the base-case

AE, adverse events; gen-gamma, generalized gamma distribution; K-M, Kaplan-Meier; OS, overall survival; PFS, progression-free survival; PS, partitioned survival; SAE, serious adverse events; ToT, time-on-treatment; TPS, tumor proportion score.

Regions & Currencies: CN, China; UK, The United Kingdom; US, The United States.

In five studies, the transition between health states was modeled over a time horizon of a lifetime or until 99% of the patients had died. A shorter time horizon of 20 years was used in 11 studies, 10 years in 7 studies, and 5 years in 1 study. The modeling cycle length was 1 week (nine studies, predominantly with the PS model), 3 weeks (nine studies, predominantly with the Markov model), 30 days (four studies), or 6 weeks (one study). The choice of the cycle lengths, however, was scarcely justified in most studies. The specific parametric models used for extrapolating the survival data were described in 20 studies. Moreover, 17 studies reported the criteria to measure the goodness-of-fit of the parametric distributions for survival extrapolation. Among these, the Akaike Information Criterion (AIC), Bayesian Information Criterion (BIC), and visual inspections were adopted in 13 studies, AIC and visual examination in 2 studies, and the coefficient of determination, R^2^, in 2 studies. Common choices of distributions included the Weibull, log-normal, log-logistic, and generalized gamma distribution. In seven studies, when modeling clinical efficacy data, in addition to PFS and OS, time-on-treatment (ToT) was considered to accurately reflect the duration of the treatment schemes. In nine studies using the PS model, survival was modeled using a piece-wise method in which the original Kaplan-Meier data or different distributions could be applied at different phases of the survival curves, with specific cutoff points described.

Among the 12 studies using the Markov model, 9 studies employed time-varying transition probabilities based on time-to-event data and 3 studies assumed fixed transition probabilities between the health states estimated from the survival data. The relevant survival functions for the calculation of transitional probabilities were provided in two studies that applied time-varying transition probabilities. Among the studies using fixed transition probabilities, specific transition probability values were provided only in one article. Half-cycle correction was reported in three studies.

#### Model parameter input

The clinical efficacy inputs in all 24 studies were derived from the PFS and OS curves of one or more RCTs. The sources of cost inputs were closely related to the research perspective of each study. Despite different perspectives across countries, only direct medical costs were considered in all studies. The commonly reported costs were drug cost, administration cost, disease management cost, adverse event (AE) management cost, and terminal care cost. Additionally, in the studies where PD-L1 expression levels were differentiated, the cost of PD-L1 testing was also considered. The estimation of subsequent treatment costs following disease progression largely depended on assumptions made about subsequent treatment regimes. In 13 studies, the proportions of patients receiving subsequent treatment and regimen details in the experimental and control groups were described separately. The AEs and related costs included in the modeling were described in 22 studies, all of which encompassed grade 3 or higher adverse events. Pneumonitis, as an AE, was additionally included in three studies according to experts’ advice because it was associated with a high management cost despite a low frequency. Discounting of both costs and outcomes in the models has been recommended by guidelines (15). In 20 studies, discounting of both costs and outcomes was described, most commonly with a 3% discount rate. In two studies, discounting was not performed in the base-case analysis. In one study, the researchers did not specify whether the reported discount rate was applied to costs or outcomes. In another study, only costs were discounted.

Inputs of utilities were mainly based on time-to-death or health states. Utility values were stratified in terms of days before patient death (e.g., a common stratification being <30 days, 30–180 days, 180–360 days, and >360 days to death) in 9 studies and terms of different health states (i.e., PFS state and PD state) in 15 studies. Furthermore, four studies distinguished the utilities between the treatment arms, and six studies considered the disutility of AEs.

### Results on cost-effectiveness

As the included 24 studies are CEA, incremental cost per QALY as ICER outcomes were reported in all studies. Although the interventions were generally categorized as pembrolizumab monotherapy or pembrolizumab plus chemotherapy, and the comparators were generally chemotherapy, there were differences in the specific strategies used for the CEA in each study. Similarly, while some studies used the same clinical trial, e.g. KN-189 as their clinical data inputs, some studies might only use a subgroup of the trial population for the CEA. In order to compare the results from different studies, the perspectives, clinical data sources, willingness-to-pay (WTP) thresholds, the corresponding treating strategies and comparator strategies, ICER per QALY, and conclusion of cost-effectiveness of the base-case analysis in each study are summarized in **Table 3**.

**Table 3:** Key information and results of base-case analysis and uncertainty analysis.

Reference	Perspective	Clinical Data Inputs	WTP threshold	Treating Strategy	Comparison Strategy	ICER (cost per QALY)	Cost-effective? (Yes/No)
Lei (16)	China society	KN189	RMB 193,500/QALY	Pembrolizumab + chemotherapy (P+C) for all patients	Platinum-based Chemotherapy (PT-C) for all patients	RMB 1,198, 092.32 (USD 173,636.57) /QALY	No
Jiang (17)	China society	KN189	USD 28,106/QALY	P+C for all patients	PT-C for all patients	USD $96,644/QALY	No
Zeng (18)	US payer	KN189	USD 130,000/QALY	P+C for all patients	PT-C for all patients	USD 194,372/QALY	US: No
Insinga (19)	US payer	KN189	USD 180,000/QALY	P+C for all patients	PT-C for all patients	USD $104,823/QALY	Yes
Wan (20)	US: payer China: payer	KN189	US: USD 100,000/QALY China: USD 27,351/QALY	P+C for all patients without determination of PD-L1 status	PT-C for all patients	USD 132,392/QALY (US) USD 92,533/QALY (CN)	US: No China: No
				P+C for patients with TPS ≥1%, PT-C for other patients	PT-C for all patients	USD 77,745/QALY (US) USD 56,768/QALY (CN)	US: Yes China: No
				P+C for patients with TPS ≥50%, PT-C for other patients	PT-C for all patients	USD 44,731/QALY (US) USD 34,388/QALY (CN)	US: Yes China: No
**Wu (** 21 **)**	US: payer China: unspecified	KN189	US: USD 150,000/QALY China: USD 29,196/QALY	P+C for patients without determination of PD-L1 status	PT-C for all patients	USD 122,248/QALY (US NSQ) USD 121,375/QALY (US SQ) USD 47,328/QALY (CN NSQ) USD 54,805/QALY (CN SQ)	US: Yes China: No
				P+C for patients with TPS ≥1%, PT-C for other patients	PT-C for all patients	USD 127,661/QALY (US NSQ) USD 121,554/QALY (US SQ) USD 54,536/QALY (CN NSQ) USD 52,719/QALY (CN SQ)	US: Yes China: No
				P+C for patients with TPS ≥50%, PT-C for other patients	PT-C for all patients	USD 143,282/QALY (US NSQ) USD 131,495/QALY (US SQ) USD 61,686/QALY (CN NSQ) USD 65,920/QALY (CN SQ)	US: Yes China: No
**Insinga (** 22 **)**	US payer	KN189	USD 180,000/QALY	P+C for all patients	PT-C for all patients	USD $86,293/QALY	Yes
**Barbier (** 23 **)**	Swiss payer	KN189, KN024	CHF 100,000/QALY	Pembrolizumab for patients with TPS ≥50%	PT-C for patients with TPS ≥50%	CHF 68,580 (USD 73,813)/QALY	Yes
**Bhadhur (** 24 **)**	Swiss payer	KN024	CHF 100,000/QALY	Pembrolizumab for patients with TPS ≥50%	PT-C for patients with TPS ≥50%	CHF 57,402 (USD 61,782)/QALY	Yes
**Hu (** 25 **)**	UK healthcare system	KN024	GBP 50,000/QALY	Pembrolizumab for patients with TPS ≥50%	PT-C for patients with TPS ≥50%	GBP 86,913 (USD 117,550)/QALY	No
**Georgieva (** 26 **)**	UK: healthcare system US: unspecified	KN024	UK: USD 42,048/QALY US: USD 100,000/QALY	Pembrolizumab for patients with TPS ≥50%, with end-of-life (EoL) adjustment (i.e. giving utility of 1 to EoL interventions). No dependency (ND) or moderate dependency (MD) between the simulated patient outcomes of the two arms was incorporated.	PT-C for patients with TPS ≥50%	USD 34,000/QALY (UK ND^1^) USD 31,000/QALY (US ND) USD 52,000/QALY (UK MD) USD 49,000/QALY (US MD)	UK: No US: Yes
				Pembrolizumab for patients with TPS ≥50% without EoL adjustment. ND or MD between the simulated patient outcomes of the two arms was incorporated.	PT-C for patients with TPS ≥50%	USD 81,000/QALY (UK ND) USD 74,000/QALY (US ND) USD 115,000/QALY (UK MD) USD 110,000/QALY (US MD)	UK: No US: Yes
**Huang (** 27 **)**	US payer	KN024	USD 171,660/QALY	Pembrolizumab for patients with TPS ≥50%	PT-C for patients with TPS ≥50%	USD 97,621/QALY	Yes
**Liao (** 28 **)**	China society	KN024	USD 26,481/QALY	Pembrolizumab for patients with TPS ≥50%	PT-C for patients with TPS ≥50%	USD 103,128/QALY	No
**Loong (** 29 **)**	Hong Kong Hospital Authority	KN024	HKD 1,017,819/QALY	Pembrolizumab for patients with TPS ≥50%	PT-C for patients with TPS ≥50%	HKD 865,189 (USD 110,922)/QALY	Yes
**Chouaid (** 30 **)**	French healthcare system	KN024	EUR 170,000/QALY	Pembrolizumab for patients with TPS ≥50%	PT-C for patients with TPS ≥50%	EUR 84,097 (USD 95,719)/QALY (SQ) EUR 78,729 (USD 89,609)/QALY (NSQ)	Yes
**Aziz (** 31 **)**	Singapore healthcare system	KN024	SGD 100,000/QALY	Pembrolizumab for patients with TPS ≥50%	PT-C for patients with TPS ≥50%	SGD 167,692 (USD 124,729)/QALY	No
**She (** 32 **)**	US payer	KN042	USD 150,000/QALY	Pembrolizumab for patients with TPS ≥50%	PT-C for patients with TPS ≥50%	USD 136 228.82/QALY	Yes
				Pembrolizumab for patients with TPS ≥20%	PT-C for patients with TPS ≥20%	USD 160 625.98/QALY	No
				Pembrolizumab for patients with TPS ≥1%	PT-C for patients with TPS ≥1%	USD 179 530.17/QALY	No
**Weng (** 33 **)**	US healthcare system	KN042	USD 180,000/QALY	Pembrolizumab for patients with TPS ≥50%	PT-C for patients with TPS ≥50%	USD 47,596/QALY	Yes
				Pembrolizumab for patients with TPS ≥20%	PT-C for patients with TPS ≥20%	USD 47,184/QALY	Yes
				Pembrolizumab for patients with TPS ≥1%	PT-C for patients with TPS ≥1%	USD 68,061/QALY	Yes
**Huang (** 34 **)**	US payer	KN042	USD 194,000/QALY	Pembrolizumab for patients with TPS ≥1%	PT-C for patients with TPS ≥1%	USD 130,155/QALY	Yes
**Zhou (** 35 **)**	China payer	KN042	USD 26,508/QALY	Pembrolizumab for patients with TPS ≥50%	PT-C for patients with TPS ≥50%	USD 36,493/QALY (TPS ≥ 50%)	No
				Pembrolizumab for patients with TPS ≥20%	PT-C for patients with TPS ≥20%	USD 42,311/QALY (TPS ≥ 20%)	No
				Pembrolizumab for patients with TPS ≥1%	PT-C for patients with TPS ≥1%	USD 39,404/QALY (TPS ≥ 1%)	No
**Xu (** 36 **)**	China healthcare system	KN042	RMB 212,676/QALY	Pembrolizumab for patients with TPS ≥50%	PT-C for patients with TPS ≥50%	RMB 395,332.25 (USD 57,295)/QALY	No
				Pembrolizumab for patients with TPS ≥20%	PT-C for patients with TPS ≥20%	RMB 735,613.67 (USD 106,611)/QALY	No
				Pembrolizumab for patients with TPS ≥1%	PT-C for patients with TPS ≥1%	RMB 597,770.09 (USD 86,633)/QALY	No
**Xu (** 37 **)**	China healthcare system	KN042	RMB 193,932/QALY	Pembrolizumab for patients with TPS ≥50%	PT-C for patients with TPS ≥50%	RMB 228,254.12 (USD 62,186)/QALY	No
				Pembrolizumab for patients with TPS ≥20%	PT-C for patients with TPS ≥20%	RMB 351,267.03 (USD 50,908) /QALY	No
				Pembrolizumab for patients with TPS ≥1%	PT-C for patients with TPS ≥1%	RMB 256,990.96 (USD 26,770)/QALY	No
**Huang (** 38 **)**	US payer	KN010	USD 171,660/QALY	Pembrolizumab for patients with TPS ≥50%	Docetaxel for patients with TPS ≥50%	USD 168,619/QALY	Yes
**Aguiar (** 39 **)**	US Medicare system	KN010	USD 100,000/QALY	Pembrolizumab for patients with TPS ≥1%	Docetaxel for patients with TPS ≥1%	USD 98,421/QALY	Yes

P+C, pembrolizumab plus chemotherapy; EoL, end-of-life; ICER, incremental cost-effectiveness ratio; KN, KEYNOTE; MD, moderate dependency; ND, no dependency; NSQ, non-squamous; PD-L1, programmed death ligand-1; PT-C, platinum-based chemotherapy; QALY, quality-adjusted life years; SQ, squamous; TPS, tumor proportion score; WTP, willingness to pay;

Regions & Currencies: CN, China; UK, The United Kingdom; US, The United States; USD, US dollar; RMB, renminbi; GBP, pound sterling; SGD, Singapore dollar; HKD, Hong Kong dollar; CHF, Swiss franc.

#### Pembrolizumab monotherapy vs. chemotherapy

Conclusions on the cost-effectiveness of pembrolizumab monotherapy compared with platinum-based chemotherapy for patients with first-line advanced, high PD-L1 expression (≥50%) NSCLC without EGFR or ALK mutations were reported in eight studies that modeled data from the KEYNOTE-024 trial. The research perspectives of these studies were based on the health system or healthcare payers of regional backgrounds, including Mainland China, Hong Kong, the United States, the United Kingdom, Switzerland, and France. The results of the cost-effectiveness analysis suggested that pembrolizumab monotherapy was more likely to be cost-effective in Switzerland, the United States, France, and Hong Kong, whereas the studies conducted in the background of the United Kingdom, mainland China, and Singapore indicated the contrary. Among these, two studies (23, 24) from the perspective of Swiss payers reached ICERs of CHF 68,580 (USD 73,813)/QALY and CHF 57,402 (USD 61,782)/QALY respectively, both below the same WTP. The UK perspective of the study by Georgieva et al. (26) explored different scenarios in the base-case, assuming different dependency levels between the outcomes of the treating arm and adjusting utility values for end-of-life interventions. When they assumed moderate dependency between the outcomes of the two arms, and without end-of-life utility adjustment, the ICER (USD 115,000/QALY) was close to that of another study [Hu et al.] with the UK healthcare system perspective (USD 117,550/QALY).

The cost-effectiveness of pembrolizumab monotherapy versus platinum-based chemotherapy for patients with first-line advanced, PD-L1 positive (≥1%) NSCLC without EGFR or ALK mutation was reported in six studies based on the KEYNOTE-042 clinical trial data. Three studies were conducted from the perspective of US third-party payers but led to rather distinct ICER estimates. Research by She et al. (32) using a Markov model with time-varying transition probabilities resulted in ICERs of $136,228.82/QALY, $160,625.98/QALY, and $179,530.17/QALY for patients with PD-L1 expression of ≥50%, ≥20%, and ≥1%, respectively. With a WTP threshold of $150,000/QALY, immunotherapy with pembrolizumab was cost-effective only for patients with PD-L1 expression ≥50%. The study by Weng et al. (33) using a Markov model with time-varying transition probabilities showed that the estimated ICER for patients with PD-L1 expression of ≥50%, ≥20%, and ≥1% were $47,596/QALY, $47,184/QALY, and $68,061/QALY, respectively. At a WTP threshold of $180,000/QALY, pembrolizumab was concluded to be cost-effective for all three groups. Huang et al. (27) conducted a base-case analysis without differentiating between the PD-L1 expression levels of patients and a subgroup analysis that distinguished between patients based on PD-L1 expression. Their base-case result showed an ICER of $130,155/QALY, suggesting cost-effectiveness at a WTP threshold of $150,000/QALY. However, further results among subgroups indicated that under the same WTP threshold, the treatment strategy was cost-effective (ICER $111,781/QALY) for patients with high PD-L1 expression (≥50%) but not for those with low expression levels (1%–49%) (ICER $161,546/QALY). Three other studies were conducted from the perspective of China’s healthcare system. In two separate reports, Xu et al. adopted the PS model (37) and Markov model (36) using the same clinical data, and the ICER values generated by the two types of models were dissimilar. The other study by Zhou et al. (35) was from a Chinese perspective and employed a Markov model; nevertheless, it produced seemingly closer ICERs than those obtained by Xu et al. (37) using the PS model. The three studies arrived at the same conclusion that the therapy was unlikely to be cost-effective in China, regardless of the patients’ TPS.

Two studies based on the KEYNOTE-010 trial data reported the cost-effectiveness of pembrolizumab monotherapy compared with docetaxel as a second-line treatment for patients with NSCLC whose PD-L1 expression was ≥1%. The study of Huang et al. (38) utilized a PS model and generated an ICER of $168,619/QALY, which was cost-effective at a WTP threshold of $171,000/QALY. Anguiar et al. (39) described a decision-analytic model without a specific type, which was applied to evaluate several immunotherapy agents. The results relevant to pembrolizumab estimated that the strategy would obtain an ICER of $98,421/QALY compared with docetaxel. The number was remarkably lower than that obtained by Huang et al. (38), despite similar conclusions that pembrolizumab was cost-effective compared to docetaxel.

#### Pembrolizumab combined with chemotherapy vs. chemotherapy

The cost-effectiveness of pembrolizumab combined with chemotherapy versus platinum-based doublet chemotherapy for the first-line treatment of patients with metastatic nonsquamous NSCLC without EGFR or ALK mutations was reported in six studies. The trial population of KEYNOTE-189 included patients with all PD-L1 expression levels. The study by Lei et al. (16) and Jiang et al. (17) investigated the same strategies from similar perspectives but different models, and arrived at ICER of approximately $173,636/QALY (estimated from RMB) and $96,644/QALY, respectively. Wan et al. (20) and Wu & Lu (21) used the similar models, perspectives and strategies. Both articles explored three different treating strategies based on the patient’s PD-L1 expression level in the US and China context. Some results were similar, but others were drastically different. For example, with the treating strategy of giving patients with PD-L1 expression ≥50% combination therapy and all other patients chemotherapy, and the comparator strategy of treating all patients with chemotherapy, the ICER by Wan et al. (20) in the US perspective was $47,328/QALY, significantly lower than the result by Wu & Lu for non-squamous NSCLC with the same strategies and perspective ($143,282/QALY). Setting three times China’s GDP (approximately $29,000 in 2018) as the WTP threshold, the results of all four studies, despite the marked variations in ICERs, agreed that this combination therapy was not cost-effective in China. From the perspective of healthcare payers in the US, the ICERs for the combination therapy compared with chemotherapy were documented in four studies. Zeng et al. (18) reported the highest ICER of $194,372/QALY by using a dynamic Markov model at a WTP threshold of $130,000/QALY. The researchers concluded that pembrolizumab combined with chemotherapy would not be cost-effective in the US. Wan et el (20). also reached a similar conclusion, with an ICER of $132,392/QALY at a WTP of $100,000/QALY. Contrarily, two more studies, one using the Markov model (21) and the other (22) the PS model, reported ICERs of $122,248/QALY and $104,823/QALY, respectively, based on a WTP threshold of $150,000/QALY. Two (20, 21) of the six studies conducted an incremental analysis of the strategies based on the PD-L1 tests in the contexts of the US and China. The findings of Wan et al. (20) showed that testing for PD-L1 decreased the ICERs drastically in both China and US when pembrolizumab plus chemotherapy was administered to patients with PD-L1 ≥1% or ≥50%, whereas chemotherapy was given to the rest. The results of Wu et al. (21), however, suggested that PD-L1 test-based treatment strategies increased the ICERs compared with the strategy that did not distinguish between the PD-L1 expression levels.

Two studies based on the KEYNOTE-407 trial data analyzed the cost-effectiveness of pembrolizumab combined with chemotherapy compared with chemotherapy for the first-line treatment of patients with squamous NSCLC without EGFR or ALK mutations. A study by Insinga et al. (19), which used the PS model, concluded that from the perspective of US healthcare insurance payers, the combination strategy without determination of PD-L1 status would produce an ICER of $86,293/QALY and be cost-effective at a WTP threshold of $100,000/QALY. In the study by Wu & Lu (21), which was conducted from a similar US perspective and employed a Markov model, the same combination strategy produced an ICER of $121,375/QALY and was found to be cost-effective under a WTP threshold of $150,000/QALY Wu & Lu (21) also explored two other PD-L1 test-based strategies for the KEYNOTE-407 population from the perspectives of both the US and China, and found that the combination strategy, regardless of the patient’s PD-L1 levels, was not cost-effective for the population with squamous NSCLC in China but cost-effective in the US.

### Uncertainty analysis

One-way sensitivity analysis was performed in all 24 studies and two-way sensitivity analysis in 3 studies, where parameter uncertainties of the models were examined. The results of one-way sensitivity analysis from different studies indicated that factors having the biggest impact on the ICER can vary greatly. The cost of ICIs was a common factor reported by several studies to exert a significant impact on the results. In 20 studies, a probability sensitivity analysis with the cost-effectiveness acceptability curve (CEAC) was performed, which estimated the acceptability for pembrolizumab regimens compared to chemotherapy at several WTP thresholds. Scenario analysis was performed in 19 studies. Frequent scenarios included alternative parametric distributions for extrapolation of survival data, alternative modeling time horizon and discount rates, utility values obtained from other sources, etc., or considering charity or aiding projects that helped in covering the cost of immunotherapeutic medications. The results of scenario analysis alluded that although most of the scenario assumptions influenced the ICERs to some extent, only two scenarios from two studies by Xu et el (36, 37). reversed the base-case conclusions (i.e., from being not cost-effective to cost-effective). These scenarios assumed a significant reduction in the drug price of pembrolizumab owing to charity projects.

## Discussion

This systematic review included 24 CEA studies that were relevant to pembrolizumab in the treatment of NSCLC. With reference to a reporting quality checklist from *The China Guidelines for Pharmacoeconomic Evaluations 2020 Edition* (15), the included studies met, on average, 78.6% of the 40 listed items, and thus, were of medium quality. There are other available checklists for health economic studies, such as the Consolidated Health Economic Evaluation Reporting Standards (CHEERS 2022) (40) and the health technology assessment guidelines by Phillips (41). Although these standard checklists are generally applicable to health economic evaluations, the China Guidelines for Pharmacoeconomic Evaluations checklist is more specifically applicable to cost-effectiveness analysis, which matches the subject of the present review. However, the Chinese guideline has its limitations and does not cover the full scope of quality assessment for cost-effectiveness analysis studies. In particular, this checklist puts on emphasis on the reporting format, instead of the quality of methodologies. Therefore, it may be interesting to compare the quality assessment results using different criteria in the future.

Analysis of the modeling methods revealed that, in most studies, several key modeling assumptions, such as health states, research perspective, modeling cycle, and time horizon, were adequately described. However, the choice of a specific model design, e.g. the Markov model with fixed transition probabilities, over other options, was insufficiently justified. Moreover, the sources of input parameters of clinical efficacy, costs, and utility values were specified in most studies. However, as certain data inputs were derived from other literature, little explanation was given on whether the data, e.g. utility values, were suitable. This omission may add to the parameter uncertainty inherent in the model assumptions and lead to conclusions that are inapplicable to the proposed perspective. Most studies were able to describe the parametric distributions used to extrapolate the survival data, but the selection of the distributions was not always justified and specific parameter values were scarcely presented.

Although the included studies were conducted from various study perspectives and country backgrounds, only direct medical costs were considered as cost inputs. Thus, it was unclear how the different perspectives (society, payers, or healthcare systems) could be reflected in the type of cost inputs. For studies that took a societal perspective, guidelines have recommended that indirect costs, e.g. time costs and opportunity costs (42), be considered in the CEA in additional to direct medical costs. To calculate some indirect cost, or instance, the use of the human capital approach (HCA) is recommended while performing calculations for the indirect cost incurred during treatment. This approach makes use of the average salary from the labor market to estimate the productivity loss caused by the disease or early death based on the assumption that all lost time will be used for prediction (43).

Analysis of incremental health economic evaluations showed that ICERs generated from different study perspectives and modeling methods can vary immensely, even when the studies had the same clinical data inputs and compared similar strategies. For example, the studies by Lei et al. (16) and Wu & Lu (21) came to drastically ICER results of USD $173,636.57/QALY and $47,328/QALY, using the same model type, same trial population and similar treating strategies. Potential factors that contribute to this drastic difference may lie in the model assumptions and parameter inputs. The discrepancy in ICER values is the direct result of the differences in their modelled total costs, LYs gained and QALYs. The difference in total costs might result from different cost data sources, cost composition, assumptions of subsequent treatments (i.e. second-line treatments). Utility values also varied greatly from different literature. The health states-based utilities in Lei’s study (16) were from a quality-of-life report for the KEYNOTE-024 trial, although the CEA was based on the KEYNOTE-189 trial. These utility values led to much lower total LYs and QALYs gained compared to the study by Wu & Lu (21), which used utility scores based on the patient’s time-to-death from published quality-of-life reports of the KEYNOTE-189 trial. We can see that there is a large uncertainty in the parameter inputs, specifically cost and utility data, used in the model-based CEA, which can largely contribute to disparate ICER results of similar studies.

Compared with the conventional standard chemotherapy, pembrolizumab combined with chemotherapy or pembrolizumab monotherapy seems more likely to be cost-effective in developed countries, such as the United States and Switzerland. In China, immunotherapy strategies involving pembrolizumab have hardly been reported to be cost-effective by relevant pharmacoeconomic studies. Also critical to the conclusions on cost-effectiveness is the willingness-to-pay (WTP) threshold defined under a certain perspective in each study. The WHO recommends the use of fewer than three times the GDP per capita of the country in question as to the threshold (44). Hence, the WTP thresholds can vary greatly depending on the country’s economic development. Pembrolizumab combined with chemotherapy has been concluded to be cost-effective compared with conventional chemotherapy in studies performed from the perspective of countries that are believed to be wealthy, such as the United States and Switzerland. It is worth noting that studies confirming the cost-effectiveness of pembrolizumab have generally set relatively high WTP thresholds. For example, in three studies by Huang et al. (27, 34, 38), the WTP threshold was set at $150,000/QALY or even higher, which, although close to three times the GDP of the US, may not be applicable in realistic settings. In contrast, for the United Kingdom, which is a developed country, the WTP threshold recommended by the National Health System (NHS) is £30,000–£50,000. Accordingly, pharmacoeconomic evaluations from the perspective of the UK’s NHS system allude that pembrolizumab is unlikely to be cost-effective. This observation agrees with the results of the studies on European reality (45, 46). Giuliani calculated the differences in OS in phase III RCTs in first-line treatments with pembrolizumab for metastatic NSCLC and the pharmacological costs necessary to derive the benefit in OS for each trial (45, 46). Pembrolizumab was found to be a cost-effective first-line treatment for patients with metastatic NSCLC.

Two published systematic reviews are similar to our study. Varma et al. (47) systematically reviewed the cost-effectiveness analyses of ICIs. Three pharmacoeconomic evaluations related to pembrolizumab in the treatment of NSCLC (25, 28, 36) were included, and pembrolizumab was found to be cost-effective only in studies conducted from the perspective of the United States. Another systematic review by Qiao et al. (48) included 14 studies that reported the cost-effectiveness of pembrolizumab in the treatment of NSCLC. Similar to the findings of our review. their research also found that the results varied greatly among the included studies, which led to completely different conclusions. Thus, for payers and policymakers, it is necessary to carefully evaluate the model design and assumptions in model-based health economic studies and to formulate policies based on strong evidence from robust studies with high validity. Compared to the two existing reviews, in addition to comparing cost-effectiveness results, our review puts an emphasis on the reporting quality of the included studies and explores the potential influence of the modeling methods on the final results. Our findings can hopefully provide meaningful directions for improving the quality of model-based cost-effectiveness analysis as well as other related pharmacoeconomic evaluations.

One limitation of our study is that we could not quantify the results through a meta-analysis. As the included cost-effectiveness studies varied in their country perspectives and sources of data, it was difficult to conduct a quantitative pooling of the results. Another limitation is that, for similar studies with disparate ICER results, we could not pinpoint the extent to which the factors (i.e. different data sources, model structures, and parameter inputs) contributed to the different results. In addition, our review only includes Chinese and English language studies, which may involve a language bias. Furthermore, the scope of the study is relatively small, as it merely considered pembrolizumab in the treatment of NSCLC patients. In the future, studies that focus on a more thorough evaluation of different types of ICIs and conditions are warranted.

### Conclusions

The present systematic review of 24 health economic studies was conducted, which revealed that the existing cost-effectiveness analysis studies are generally of moderate reporting quality and that the modeling methods used in these studies need to be further strengthened for efficacy, in order to achieve valid outcomes to better support relevant policy decision-making. The cost-effectiveness of pembrolizumab therapy strategies for NSCLC varies across countries, highlighting the need to pay more attention to the methodologies of pharmacoeconomic research to produce correct results of cost-effectiveness across countries. Future research may focus on a more systematic, quantitative approach to comprehensively evaluate the reporting quality and results of studies.

## Author contributions

CZ: Literature search, Study selection, Data extraction and quality assessment, Writing -Review and Editing. JZ: Literature search, Study selection, Data extraction and quality assessment, Writing - Original Draft. JT: Writing -Review and Editing. PT: Formulation or evolution of overarching research aims, Writing -Review and Editing. WL: Ideas, Formulation or evolution of overarching research aims, Supervision and Project administration, Final approval of the version to be submitted. All authors contributed to the article and approved the submitted version.

## Conflict of interest

The authors declare that the research was conducted in the absence of any commercial or financial relationships that could be construed as a potential conflict of interest.

## Publisher’s note

All claims expressed in this article are solely those of the authors and do not necessarily represent those of their affiliated organizations, or those of the publisher, the editors and the reviewers. Any product that may be evaluated in this article, or claim that may be made by its manufacturer, is not guaranteed or endorsed by the publisher.

## References

[1] Sung H Ferlay J Siegel RL Laversanne M Soerjomataram I Jemal A . Global cancer statistics 2020: Globocan estimates of incidence and mortality worldwide for 36 cancers in 185 countries. CA Cancer J Clin (2021) 71(3):209–49. doi: 10.3322/caac.21660 33538338

[2] Sher T Dy GK Adjei AA . Small cell lung cancer. Mayo Clin Proc (2008) 83(3):355–67. doi: 10.4065/83.3.355 18316005

[3] Ho C Tong KM Ramsden K Ionescu DN Laskin J . Histologic classification of non-Small-Cell lung cancer over time: Reducing the rates of not-Otherwise-Specified. Curr Oncol (2015) 22(3):e164–70. doi: 10.3747/co.22.2339 PMC4462538 26089727

[4] Rosell R Karachaliou N . Large-Scale screening for somatic mutations in lung cancer. Lancet (2016) 387(10026):1354–6. doi: 10.1016/s0140-6736(15)01125-3 26777918

[5] Socinski MA Bondarenko I Karaseva NA Makhson AM Vynnychenko I Okamoto I . Weekly nab-paclitaxel in combination with carboplatin versus solvent-based paclitaxel plus carboplatin as first-line therapy in patients with advanced non-small-cell lung cancer: Final results of a phase iii trial. J Clin Oncol (2012) 30(17):2055–62. doi: 10.1200/jco.2011.39.5848 22547591

[6] Vaddepally RK Kharel P Pandey R Garje R Chandra AB . Review of indications of fda-approved immune checkpoint inhibitors per nccn guidelines with the level of evidence. Cancers (Basel) (2020) 12(3):738. doi: 10.3390/cancers12030738 PMC7140028 32245016

[7] Herbst RS Baas P Kim DW Felip E Pérez-Gracia JL Han JY . Pembrolizumab versus docetaxel for previously treated, pd-L1-positive, advanced non-Small-Cell lung cancer (Keynote-010): A randomised controlled trial. Lancet (2016) 387(10027):1540–50. doi: 10.1016/s0140-6736(15)01281-7 26712084

[8] Reck M Rodríguez-Abreu D Robinson AG Hui R Csőszi T Fülöp A . Pembrolizumab versus chemotherapy for pd-L1-positive non-small-cell lung cancer. N Engl J Med (2016) 375(19):1823–33. doi: 10.1056/NEJMoa1606774 27718847

[9] Langer CJ Gadgeel SM Borghaei H Papadimitrakopoulou VA Patnaik A Powell SF . Carboplatin and pemetrexed with or without pembrolizumab for advanced, non-squamous non-small-cell lung cancer: A randomised, phase 2 cohort of the open-label keynote-021 study. Lancet Oncol (2016) 17(11):1497–508. doi: 10.1016/s1470-2045(16)30498-3 PMC6886237 27745820

[10] Gandhi L Rodríguez-Abreu D Gadgeel S Esteban E Felip E De Angelis F . Pembrolizumab plus chemotherapy in metastatic non-small-cell lung cancer. N Engl J Med (2018) 378(22):2078–92. doi: 10.1056/NEJMoa1801005 29658856

[11] Paz-Ares L Luft A Vicente D Tafreshi A Gümüş M Mazières J . Pembrolizumab plus chemotherapy for squamous non-small-cell lung cancer. N Engl J Med (2018) 379(21):2040–51. doi: 10.1056/NEJMoa1810865 30280635

[12] Paz-Ares LG Luft A Tafreshi A Gumus M Mazieres J Hermes B . Phase 3 study of carboplatin-Paclitaxel/Nab-Paclitaxel (Chemo) with or without pembrolizumab (Pembro) for patients (Pts) with metastatic squamous (Sq) non-small cell lung cancer (Nsclc). J Clin Oncol (2018) 36(15_suppl):105. doi: 10.1200/JCO.2018.36.15_suppl.105

[13] Mok TSK Wu YL Kudaba I Kowalski DM Cho BC Turna HZ . Pembrolizumab versus chemotherapy for previously untreated, pd-L1-expressing, locally advanced or metastatic non-small-cell lung cancer (Keynote-042): A randomised, open-label, controlled, phase 3 trial. Lancet (2019) 393(10183):1819–30. doi: 10.1016/s0140-6736(18)32409-7 30955977

[14] Page MJ McKenzie JE Bossuyt PM Boutron I Hoffmann TC Mulrow CD . The prisma 2020 statement: An updated guideline for reporting systematic reviews. Bmj (2021) 372:n71. doi: 10.1136/bmj.n71 33782057 PMC8005924

[15] Liu G Hu S Wu J Wu J Dong Z Li H . China Guidelines for pharmacoeconomic evaluations. 1st Ed. Beijing: China Market Press (2020).

[16] Lei W Du B Lin X Huang H Liu T Chen J . Cost-effectiveness analysis on pembrolizumab combined with chemotherapy in the first-line treatment of advanced non-small cell lung cancer. Eval Anal Drug-Use Hospitals China (2020) 20(10):1208–11. doi: 10.14009/j.issn.1672-2124.2020.10.015

[17] Jiang Y Wang X . Cost-effectiveness analysis of pembrolizumab plus standard chemotherapy versus chemotherapy alone for first-line treatment of metastatic non-squamous non-small-cell lung cancer in China. Eur J Hosp Pharm (2020) 29(3):139–144. doi: 10.1136/ejhpharm-2020-002208 PMC9047884 32737070

[18] Zeng X Wan X Peng L Peng Y Ma F Liu Q . Cost-effectiveness analysis of pembrolizumab plus chemotherapy for previously untreated metastatic non-small cell lung cancer in the USA. BMJ Open (2019) 9(12):e031019. doi: 10.1136/bmjopen-2019-031019 PMC6924863 31831534

[19] Insinga RP Vanness DJ Feliciano JL Vandormael K Traore S Burke T . Cost-effectiveness of pembrolizumab in combination with chemotherapy in the 1st line treatment of non-squamous nsclc in the us. J Med Econ (2018) 21(12):1191–205. doi: 10.1080/13696998.2018.1521416 30188231

[20] Wan N Zhang TT Hua SH Lu ZL Ji B Li LX . Cost-effectiveness analysis of pembrolizumab plus chemotherapy with pd-L1 test for the first-line treatment of nsclc. Cancer Med (2020) 9(5):1683–93. doi: 10.1002/cam4.2793 PMC7050096 31945265

[21] Wu B Lu S . The effect of pd-L1 categories-directed pembrolizumab plus chemotherapy for newly diagnosed metastatic non-small-cell lung cancer: A cost-effectiveness analysis. Transl Lung Cancer Res (2020) 9(5):1770–84. doi: 10.21037/tlcr-19-605 PMC7653112 33209600

[22] Insinga RP Vanness DJ Feliciano JL Vandormael K Traore S Ejzykowicz F . Cost-effectiveness of pembrolizumab in combination with chemotherapy versus chemotherapy and pembrolizumab monotherapy in the first-line treatment of squamous non-small-cell lung cancer in the us. Curr Med Res Opin (2019) 35(7):1241–56. doi: 10.1080/03007995.2019.1571297 30649973

[23] Barbier MC Pardo E Panje CM Gautschi O Lupatsch JE . A cost-effectiveness analysis of pembrolizumab with or without chemotherapy for the treatment of patients with metastatic, non-squamous non-small cell lung cancer and high pd-L1 expression in Switzerland. Eur J Health Econ (2021) 22(5):669–77. doi: 10.1007/s10198-021-01282-4 PMC8214587 33745093

[24] Bhadhuri A Insinga R Guggisberg P Panje C Schwenkglenks M . Cost effectiveness of pembrolizumab vs chemotherapy as first-line treatment for metastatic nsclc that expresses high levels of pd-L1 in Switzerland. Swiss Med Wkly (2019) 149:w20170. doi: 10.4414/smw.2019.20170 31880807

[25] Hu X Hay JW . First-line pembrolizumab in pd-L1 positive non-small-cell lung cancer: A cost-effectiveness analysis from the uk health care perspective. Lung Cancer (2018) 123:166–71. doi: 10.1016/j.lungcan.2018.07.012 30089590

[26] Georgieva M da Silveira Nogueira Lima JP Aguiar P Jr. de Lima Lopes G Jr. Haaland B . Cost-effectiveness of pembrolizumab as first-line therapy for advanced non-small cell lung cancer. Lung Cancer (2018) 124:248–54. doi: 10.1016/j.lungcan.2018.08.018 30268469

[27] Huang M Lou Y Pellissier J Burke T Liu FX Xu R . Cost effectiveness of pembrolizumab vs. standard-of-Care chemotherapy as first-line treatment for metastatic nsclc that expresses high levels of pd-L1 in the united states. Pharmacoeconomics (2017) 35(8):831–44. doi: 10.1007/s40273-017-0527-z PMC5548835 28620848

[28] Liao W Huang J Hutton D Li Q . Cost-effectiveness analysis of first-line pembrolizumab treatment for pd-L1 positive, non-small cell lung cancer in China. J Med Econ (2019) 22(4):344–9. doi: 10.1080/13696998.2019.1570221 30646794

[29] Loong HH Wong CKH Leung LKS Dhankhar P Insinga RP Chandwani S . Cost effectiveness of pd-L1-based test-and-treat strategy with pembrolizumab as the first-line treatment for metastatic nsclc in Hong Kong. Pharmacoecon Open (2020) 4(2):235–47. doi: 10.1007/s41669-019-00178-7 PMC7248157 31531842

[30] Chouaid C Bensimon L Clay E Millier A Levy-Bachelot L Huang M . Cost-effectiveness analysis of pembrolizumab versus standard-of-care chemotherapy for first-line treatment of pd-L1 positive (>50%) metastatic squamous and non-squamous non-small cell lung cancer in France. Lung Cancer (2019) 127:44–52. doi: 10.1016/j.lungcan.2018.11.008 30642550

[31] Aziz MIA Tan LE Tan WHG Toh CK Loke LPY Pearce F . Cost-effectiveness analysis of pembrolizumab monotherapy versus chemotherapy for previously untreated advanced non-small cell lung cancer. J Med Econ (2020) 23(9):952–60. doi: 10.1080/13696998.2020.1775620 32462958

[32] She L Hu H Liao M Xia X Shi Y Yao L . Cost-effectiveness analysis of pembrolizumab versus chemotherapy as first-line treatment in locally advanced or metastatic non-small cell lung cancer with pd-L1 tumor proportion score 1% or greater. Lung Cancer (2019) 138:88–94. doi: 10.1016/j.lungcan.2019.10.017 31655368

[33] Weng X Luo S Lin S Zhong L Li M Xin R . Cost-utility analysis of pembrolizumab versus chemotherapy as first-line treatment for metastatic non-small cell lung cancer with different pd-L1 expression levels. Oncol Res (2020) 28(2):117–25. doi: 10.3727/096504019x15707883083132 PMC7851532 31610828

[34] Huang M Lopes GL Insinga RP Burke T Ejzykowicz F Zhang Y . Cost-effectiveness of pembrolizumab versus chemotherapy as first-line treatment in pd-L1-Positive advanced non-small-cell lung cancer in the usa. Immunotherapy (2019) 11(17):1463–78. doi: 10.2217/imt-2019-0178 31738117

[35] Zhou K Jiang C Li Q . Cost-effectiveness analysis of pembrolizumab monotherapy and chemotherapy in the non-small-cell lung cancer with different pd-L1 tumor proportion scores. Lung Cancer (2019) 136:98–101. doi: 10.1016/j.lungcan.2019.08.028 31476529

[36] Xu HM A . Cost-effectiveness analysis of pembrolizumab versus chemotherapy as first-line treatment in non-small cell lung cancer in China based on Markov model. Chin J Mod Appl Pharm (2021) 38(04):473–9. doi: 10.13748/j.cnki.issn1007-7693.2021.04.016

[37] Xu HM A . Cost-effectiveness analysis of pembrolizumab versus chemotherapy as first-line treatment in non-small cell lung cancer with different pd-L1 expression levels based on partitioned survival model. Chin J Hosp Pharm (2020) 40(23):2468–73. doi: 10.13286/j.1001-5213.2020.23.14

[38] Huang M Lou Y Pellissier J Burke T Liu FX Xu R . Cost-effectiveness of pembrolizumab versus docetaxel for the treatment of previously treated pd-L1 positive advanced nsclc patients in the united states. J Med Econ (2017) 20(2):140–50. doi: 10.1080/13696998.2016.1230123 27571538

[39] Aguiar PN Jr. Perry LA Penny-Dimri J Babiker H Tadokoro H de Mello RA . The effect of pd-L1 testing on the cost-effectiveness and economic impact of immune checkpoint inhibitors for the second-line treatment of nsclc. Ann Oncol (2017) 28(9):2256–63. doi: 10.1093/annonc/mdx305 28633409

[40] Husereau D Drummond M Petrou S Carswell C Moher D Greenberg D . Consolidated health economic evaluation reporting standards (CHEERS) statement. Value Health (2013) 16(2):e1–5. doi: 10.1007/s40273-013-0032-y 23538200

[41] Philips Z Bojke L Sculpher M Claxton K Golder S . Good practice guidelines for decision-analytic modelling in health technology assessment. PharmacoEconomics (2006) 24(4):355–71. doi: 10.2165/00019053-200624040-00006 16605282

[42] Garrison LP Mansley EC Abbott TA Bresnahan BW Hay JW Smeeding J . Good research practices for measuring drug costs in cost-effectiveness analyses: a societal perspective: the ISPOR drug cost task force report–part II. VALUE Health (2010) 13(1):8–13. doi: 10.1111/j.1524-4733.2009.00660.x 19883405

[43] Brouwer W BF Koopmanschap MA . How to calculate indirect costs in economic evaluations. Pharmacoeconomics (1998) 13(5):563–6. doi: 10.2165/00019053-199813050-00008 10180754

[44] McDougall JA Furnback WE Wang BCM Mahlich J . Understanding the global measurement of willingness to pay in health. J Mark Access Health Policy (2020) 8(1):1717030. doi: 10.1080/20016689.2020.1717030 32158523 PMC7048225

[45] Giuliani J Bonetti A . Immunotherapy in first-line for advance non-small cell lung cancer: a cost-effective choice? Recenti Prog Med (2019) 110:138–43. doi: 10.1701/3132.31141. 30968854

[46] Giuliani J Bonetti A . Financial toxicity and non-small cell lung cancer treatment: The optimization in the choice of immune check point inhibitors. Anticancer Res (2019) 39:3961–5. doi: 10.15226/2374-6890/3/1/00143 31262928

[47] Verma V Sprave T Haque W Simone CB 2nd Chang JY Welsh JW . A systematic review of the cost and cost-effectiveness studies of immune checkpoint inhibitors. J Immunother Cancer (2018) 6(1):128. doi: 10.1186/s40425-018-0442-7 PMC6251215 30470252

[48] Qiao N Insinga R de Lima Lopes Junior G Cook J Sénécal M . A review of cost-effectiveness studies of pembrolizumab regimens for the treatment of advanced non-small cell lung cancer. Pharmacoecon Open (2021) 5(3):365–83. doi: 10.1007/s41669-020-00255-2 PMC8333166 33469803

