# Supplementary material for: Cost-Effectiveness of Pembrolizumab for the treatment of Non–Small-Cell lung cancer: A systematic review
Source: Front Oncol. 2022 Aug 26;12:815587. doi: 10.3389/fonc.2022.815587 (PMC9469648; doi:10.3389/fonc.2022.815587)
Supplement: Supplementary file 1 [file DataSheet_1.doc]

Appendix A.1. Searching strategies

1. Searching Strategies for all databases except EBSCO EconLit

| #1 | non-small cell lung cancer |
| --- | --- |
| #2 | non-small cell carcinoma |
| #3 | lung cancer |
| #4 | lung carcinoma |
| #5 | #1 OR #2 OR #3 OR #4 |
| #6 | ‘pembrolizumab’ OR 'keytruda' OR 'PD-1' OR 'programmed death 1' OR 'lambrolizumab' OR 'MK-3475' |
| #7 | #5 AND #6 |
| #8 | cost effectiveness |
| #9 | cost effectiveness analysis |
| #10 | cost of illness |
| #11 | cost benefit analysis |
| #12 | cost utility analysis |
| #13 | health care cost |
| #14 | health care financing |
| #15 | economic evaluation |
| #16 | economic aspect |
| #17 | health economics |
| #18 | #8 OR #9 OR #10 OR #11 OR #12 OR #13 OR #14 OR #15 OR #16 OR #17 |
| #19 | #7 AND #18 |

1. Searching strategies for EBSCO EconLit

“Pembrolizumab” OR “programmed death 1” OR “PD-1” OR “immunotherapy” OR “immune checkpoint inhibitors”.

Appendix A.2. Reporting Quality Checklist

| Sections | No. | Assessment questions |
| --- | --- | --- |
| Title | 1 | Does title specify the study as pharmacoeconomic research, e.g. as economic evaluation or cost-effectiveness analysis? |
|  | 2 | Are interventions described in the title? |
|  | 3 | Is the disease or health condition of question described? |
| Funding/Sponsorship | 4 | Is the source of funds/sponsorship specified? |
|  | 5 | If the potential intervention of funding/sponsorship in the study specified? |
| Abstract | 6 | Does the abstract outline the research background, objectives, methods, results, limitations, and conclusions? |
| Background | 7 | Is background information of the health condition described? |
|  | 8 | Is the basic information of the intervention(s) described? |
|  | 9 | Is a systematic literature review provided? |
|  | 10 | Is there a description of the limitations of the existing literature? |
|  | 11 | Is the research question specified? |
|  | 12 | Is the audience of the research specified? |
| Methods | 13 | Are the characteristics of the target population described? |
|  | 14 | If the research perspective specified? |
|  | 15 | Is the choice of comparators justified? |
|  | 16 | If the model’s time horizon specified? |
|  | 17 | Is there a description of the study being model-based research or research based on individual-level data? |
|  | 18 | Are the research assumptions described? |
|  | 19 | Are the components of costs specified? |
|  | 20 | Are the sources of costs specified? |
|  | 21 | Is the type of outcomes specified, i.e. whether effect, utility, or benefit is used? |
|  | 22 | Are the sources of outcome data specified? |
|  | 23 | Is the discount rate of costs specified (if required)? |
|  | 24 | If the discount rate of outcomes specified (if required)? |
|  | 25 | Is the choice of the model(s) justified? |
|  | 26 | Is there a diagram of the model structure provided? |
|  | 27 | Is there an analysis in terms of different subgroups? |
|  | 28 | Are uncertainties analyzed? |
|  | 29 | Is there a probabilistic sensitivity analysis performed? |
| Results | 30 | Are the results of key variables reported by groups? |
|  | 31 | Are the costs and outcomes results reported by groups? |
|  | 32 | Is there an incremental analysis performed? |
|  | 33 | Is the incremental cost-effectiveness ratio reported? |
|  | 34 | Is the sensitivity analysis of the main results reported? |
|  | 35 | Is the fairness impact of research results reported? |
| Discussion | 36 | Is there a discussion on the generalizability of research results? |
|  | 37 | Is there a discussion of research limitations? |
| Conclusion | 38 | Are the main findings summarized? |
| References | 39 | Are references listed? |
| Appendix | 40 | Are key data tables/figures provided in the appendix? |

Appendix A.3. Results of reporting quality checking

| **Areas** | Title | | | F/S | | A | B | | LR | | O | | Methods | | | | | | | | | | | | | | | | | Result | | | | | | D | | C | R | X |  |
| --- | --- | --- | --- | --- | --- | --- | --- | --- | --- | --- | --- | --- | --- | --- | --- | --- | --- | --- | --- | --- | --- | --- | --- | --- | --- | --- | --- | --- | --- | --- | --- | --- | --- | --- | --- | --- | --- | --- | --- | --- | --- |
| **Reference** | 1 | 2 | 3 | 4 | 5 | 6 | 7 | 8 | 9 | 10 | 11 | 12 | 13 | 14 | 15 | 16 | 17 | 18 | 19 | 20 | 21 | 22 | 23 | 24 | 25 | 26 | 27 | 28 | 29 | 30 | 31 | 32 | 33 | 34 | 35 | 36 | 37 | 38 | 39 | 40 |  |
| Lei^[16]^ | √ | √ | √ |  |  | √ |  | √ |  | √ | √ |  | √ | √ |  | √ | √ | √ | √ | √ | √ | √ |  |  |  | √ |  | √ |  | √ | √ | √ | √ | √ |  |  |  | √ | √ |  | 25 |
| Jiang^[17]^ | √ | √ | √ | √ |  | √ | √ | √ |  |  |  |  | √ | √ |  | √ | √ | √ | √ | √ | √ | √ | √ | √ | √ | √ | √ | √ | √ | √ | √ | √ | √ | √ | √ | √ | √ | √ | √ | √ | 34 |
| Zeng^[18]^ | √ | √ | √ | √ |  | √ | √ | √ |  |  | √ |  |  | √ |  | √ | √ | √ | √ | √ | √ | √ | √ | √ |  | √ | √ | √ | √ | √ | √ | √ | √ | √ |  | √ | √ | √ | √ | √ | 32 |
| Insinga^[19]^ | √ | √ | √ | √ |  | √ | √ | √ |  |  |  |  | √ | √ |  | √ | √ | √ | √ | √ | √ | √ | √ | √ | √ | √ | √ | √ | √ | √ | √ | √ | √ | √ | √ | √ | √ | √ | √ | √ | 34 |
| Wan^[20]^ | √ | √ | √ | √ |  | √ | √ | √ |  |  |  |  | √ | √ |  | √ | √ | √ | √ |  | √ | √ | √ | √ |  | √ |  | √ | √ | √ | √ | √ | √ | √ |  |  | √ | √ | √ | √ | 29 |
| Wu^[21]^ | √ | √ | √ | √ |  | √ | √ | √ |  |  |  |  | √ | √ |  | √ | √ | √ | √ | √ | √ | √ | √ | √ |  | √ | √ | √ | √ | √ | √ | √ | √ | √ | √ |  | √ | √ | √ | √ | 32 |
| Insinga^[22]^ | √ | √ | √ | √ |  | √ | √ | √ |  |  |  |  | √ | √ |  | √ | √ | √ | √ | √ | √ | √ | √ | √ | √ | √ | √ | √ | √ | √ | √ | √ | √ | √ | √ | √ | √ | √ | √ | √ | 34 |
| Barbier^[23]^ | √ | √ | √ | √ |  | √ | √ | √ |  |  |  |  | √ | √ |  | √ | √ | √ | √ |  | √ | √ | √ | √ |  |  |  | √ | √ | √ | √ | √ | √ | √ | √ |  | √ | √ | √ | √ | 29 |
| Bhadhur^[24]^ | √ | √ | √ | √ |  | √ | √ | √ |  |  | √ |  | √ | √ | √ | √ | √ | √ | √ | √ | √ | √ | √ | √ |  | √ |  | √ | √ | √ | √ | √ | √ | √ |  | √ | √ | √ | √ | √ | 32 |
| Hu^[25]^ | √ | √ | √ | √ |  | √ |  | √ |  | √ |  |  |  | √ | √ | √ | √ | √ | √ | √ | √ | √ | √ | √ |  |  |  | √ | √ | √ | √ | √ | √ | √ |  | √ | √ | √ | √ | √ | 30 |
| Georgieva^[26]^ | √ | √ | √ | √ |  | √ |  |  |  |  |  |  | √ | √ |  | √ | √ | √ | √ | √ | √ | √ | √ | √ |  | √ |  | √ | √ | √ | √ | √ | √ | √ |  | √ | √ | √ | √ | √ | 29 |
| Huang^[27]^ | √ | √ | √ | √ |  | √ | √ | √ |  |  |  | √ | √ | √ |  | √ | √ | √ | √ | √ | √ | √ | √ | √ |  | √ |  | √ | √ | √ | √ | √ | √ | √ |  | √ | √ | √ | √ | √ | 32 |
| Liao^[28]^ | √ | √ | √ | √ |  | √ | √ | √ |  | √ |  |  | √ | √ |  | √ | √ | √ | √ |  | √ | √ | √ | √ |  | √ |  | √ | √ | √ | √ | √ | √ | √ |  |  | √ | √ | √ | √ | 30 |
| Loong^[29]^ | √ | √ | √ | √ |  | √ | √ | √ |  |  |  |  |  | √ |  | √ | √ | √ | √ | √ | √ | √ | √ | √ |  | √ |  | √ | √ | √ | √ | √ | √ | √ |  | √ | √ | √ | √ | √ | 29 |
| Chouaid^[30]^ | √ | √ | √ | √ |  | √ | √ | √ |  |  |  |  | √ | √ |  | √ | √ | √ | √ | √ | √ | √ | √ | √ | √ | √ |  | √ | √ | √ | √ | √ | √ | √ | √ | √ | √ | √ | √ | √ | 33 |
| Aziz^[31]^ | √ | √ | √ | √ |  | √ | √ | √ |  | √ |  | √ | √ | √ | √ | √ | √ | √ | √ | √ | √ | √ | √ | √ |  | √ |  | √ | √ | √ | √ | √ | √ | √ | √ | √ | √ | √ | √ | √ | 35 |
| She^[32]^ | √ | √ | √ | √ |  | √ | √ | √ |  |  | √ |  |  | √ | √ | √ | √ | √ | √ | √ | √ | √ | √ | √ |  |  |  | √ | √ | √ | √ | √ | √ | √ |  | √ | √ | √ | √ | √ | 31 |
| Weng^[33]^ | √ | √ | √ | √ |  | √ | √ | √ |  | √ |  |  | √ | √ |  | √ | √ | √ | √ | √ | √ | √ | √ |  |  | √ |  | √ | √ | √ | √ | √ | √ | √ | √ |  | √ | √ | √ | √ | 31 |
| Huang^[34]^ | √ | √ | √ | √ | √ | √ | √ | √ |  |  |  | √ | √ | √ |  | √ | √ | √ | √ | √ | √ | √ | √ | √ | √ | √ | √ | √ | √ | √ | √ | √ | √ | √ | √ | √ | √ | √ | √ |  | 35 |
| Zhou^[35]^ | √ | √ | √ | √ |  | √ | √ |  |  |  |  |  |  | √ |  | √ | √ | √ | √ | √ | √ | √ | √ | √ |  |  |  | √ |  | √ | √ | √ | √ | √ |  | √ | √ | √ | √ | √ | 27 |
| Xu^[36]^ | √ | √ | √ |  |  | √ | √ | √ |  |  |  |  | √ | √ | √ | √ | √ | √ | √ | √ | √ | √ | √ | √ | √ | √ |  | √ | √ | √ | √ | √ | √ | √ |  |  | √ |  | √ | √ | 30 |
| Xu^[37]^ | √ | √ | √ | √ |  | √ | √ | √ |  |  |  |  | √ | √ |  | √ | √ | √ | √ | √ | √ | √ | √ | √ | √ | √ |  | √ | √ | √ | √ | √ | √ | √ |  |  | √ |  | √ |  | 29 |
| Huang^[38]^ | √ | √ | √ | √ | √ | √ | √ | √ |  |  |  |  | √ | √ |  | √ | √ | √ | √ | √ | √ | √ | √ | √ | √ | √ |  | √ | √ | √ | √ | √ | √ | √ |  | √ | √ | √ | √ |  | 32 |
| Aguiar^[39]^ | √ | √ | √ |  |  | √ | √ | √ |  |  |  |  |  | √ |  | √ | √ | √ | √ |  | √ | √ | √ |  |  | √ |  | √ | √ | √ | √ | √ | √ | √ |  |  | √ |  | √ |  | 24 |

Abbreviations: F/S, funding/sponsorship; A, abstract; B, research background; LR, literature review; O, research objectives; D, discussion; C, conclusion; R, references/bibliography; X, appendix.

Note: Items marked with a check represent satisfaction of the corresponding requirements, whereas blank cells represent that either the requirement was not met or the question did not apply
